# Investigation of the Potential Correlation Between RNA-Binding Proteins in the Evolutionarily Conserved MEX3 Family and Non-small-Cell Lung Cancer

**DOI:** 10.1007/s12033-022-00638-2

**Published:** 2022-12-12

**Authors:** Ming Zhang, Linfeng Cao, Gouxin Hou, Xiaodong Lv, Jingjing Deng

**Affiliations:** 1 Department of Pulmonary and Critical Care Medicine, The First Hospital of Jiaxing, Affiliated Hospital of Jiaxing University, No. 1882 South Zhonghuan Road, Jiaxing, 314000 Zhejiang People’s Republic of China; 2 Department of Oncology, The First Hospital of Jiaxing, Affiliated Hospital of Jiaxing University, Jiaxing, People’s Republic of China

**Keywords:** MEX3 family, RNA-binding proteins, Investigation, Potential correlation, Non-small-cell lung cancer

## Abstract

Members of the MEX3 (muscle excess 3) family, uniquely characterised as mRNA binding proteins, play emerging roles in the post-transcriptional regulation of programmed biological processes, including tumour cell death and immune mechanisms, and have been shown to be involved in a variety of diseases. However, the role of MEX3 in non-small cell lung cancer (NSCLC) has not been fully elucidated. In this study, we found no significant changes in the sequence and copy number of the MEX3 gene through analysis using the COSMIC database, revealing its stability during malignancy development. Its expression in NSCLC was examined using the Oncomine™ database, and the prognosis of each member gene was analysed by Kaplan–Meier. The results showed that overexpression of MEX3A, MEX3B, MEX3C and MEX3D was associated with significantly worse OS in patients with LUAD, while overexpression of MEX3D was also associated with significantly worse OS in patients with LUSC. Afterwards, we applied the Tumour Immunology Estimation Resource (TIMER) tool to assess the correlation between different MEX3 and infiltrative immune cell infiltration. Ultimately, we found that most MEX3 members were highly expressed in NSCLC, with high expression suggesting poor prognosis and correlating with immune cell infiltration. The complexity and heterogeneity of NSCLC was understood through MEX3, setting the framework for the prognostic impact of MEX3 in NSCLC patients and the development of new targeted therapeutic strategies in the future.

**Supplementary Information:**

The online version contains supplementary material available at 10.1007/s12033-022-00638-2.

## Introduction

RNA-binding proteins (RBPs) are highly conserved across species, and they function to maintain gene expression homeostasis and to regulate almost all RNA posttranscriptional processes [1–3]. RBPs act as translation inhibitors that stimulate its post-translationally modified physiologically or pathologically bound mRNA during transport, regulate mRNA stability, RNA processing, splicing, localization, export, and translation at the post-transcriptional level [4], further regulating cell differentiation and carcinogenesis [5]. Therefore, at the subcellular level, the control of protein synthesis may be a way to develop new treatment strategies for cancer [6].

The human MEX3 (muscle excess 3) family is part of the evolutionarily conserved RBP family and comprises four members (MEX3A-D) that encode different phosphorylated proteins and exhibit different expression patterns [7, 8]. Each human MEX3 protein has two K homology (KH) domains that bind RNA, and these proteins are distinguished from other RBPs by a C-terminal RING domain called the ubiquitin E3 ligase RING (Really Interesting New Gene) domain [7, 9]. The presence of this particular domain indicates that MEX3 proteins play vital roles in the balance between self-renewal and differentiation by mediating self-ubiquitination or the ubiquitination of target proteins and promoting the RING-dependent degradation of HLA-A2 (human leukocyte antigen serotype A2) mRNA [9, 10]. MEX3 proteins not only interact with different RNA sequences but also exert diverse mechanisms enabled by the RING domain; despite the increasing complexity of regulation, there is little evidence suggesting that these proteins have redundant activity [11].

Consistent with the idea of cancer as a multi-pathway disease and the multiple roles of MEX3 in regulating gene expression, MEX3 is involved in multiple biological processes in the occurrence and development of cancer [12]. MEX3 mediates cancer cell proliferation, migration, tumour immune escape mechanisms, and transcription level changes in different cancer types [13–15]; according to the tumour type and MEX3 family member, the expression of MEX3 is related to an increase or decrease in patient survival, and expression was obtained by detecting MEX3 mRNA [16–18]

However, there have been few studies [19] on the correlation between the MEX3 family and lung cancer, and the conclusions are limited. Therefore, we investigated a large sample of databases to explore MEX3 family expression, prognostic value, and immune-related effects in non-small-cell lung cancer (NSCLC), thereby providing further insights into tumour heterogeneity and as potential targets for immunotherapy [12, 20, 21].

## Materials and Methods

### Somatic Mutations in MEX3 in Lung Cancer

We investigated *MEX3* somatic mutations using the COSMIC database [22], a free online authoritative resource that provides information on gene mutations, fusions, genome rearrangements, and copy number variations in human cancer. Data for this study are from COSMIC v92 version, with a date cut-off of February 20, 2021.

### Expression of MEX3 in NSCLC

Oncomine™ 4.5 Research Edition [23] (http://www.oncomine.org/), a web-based database that contains cancer microarray datasets, was used to explore *MEX3* expression, to conduct genome-wide analyses comparing major types of cancer and normal tissues, and to compare transcriptome expression. This database currently contains 715 datasets (86,733 samples). In this study, we used the database to determine the mRNA expression of MEX3 in NSCLC and compared the mRNA levels in lung cancer patients and healthy controls with the following criteria: *P* = 0.05, fold change > 1.5, and in the top 10% of genes. Data entries from October 2020 to February 2021 were included in the analysis, and the results were visualized with GraphPad Prism 7 software (GraphPad Software, Inc.).

### MEX3 Prognostic Analysis

Kaplan–Meier plotter [24, 25] was used to assess the prognostic relevance of MEX3A-D expression in NSCLC samples. In our study, Affymetrix Identity (Jetset best Probe [26], as shown in Table 1) was used to identify available genes, and the median gene expression values were applied to divide patient samples into the high and low expression groups. In the analysis, the 95% confidence interval (CI), log-rank *P* value and hazard ratio (HR) were calculated, and "Array quality control" and "Exclude biased array" were selected to obtain numerical results through univariate Cox regression analysis. Finally, a valuable Kaplan–Meier survival curve (OS, overall survival) was generated according to these parameters.

**Table 1:** Affymetrix ID of MEX3 family in the Kaplan–Meier plotter

MEX3	Affymetrix ID (Jetset best probe set)
MEX3A	226346_at
MEX3B	223627_at
MEX3C	218247_s_at
MEX3D	91816_f_at

*MEX3* muscle excess 3

### Immunological Correlation with MEX3

The correlation of MEX3 with the immune system was evaluated through the Tumour Immune Estimation Resource (TIMER 2.0, timer.comp-genomics.org/), a web server for the comprehensive analysis of tumour-infiltrating immune cells [27]; this resource includes The Cancer Genome Atlas (TCGA) cancer genome maps from 32 cancer types, involving a total of 10,897 samples, and returns data on six subsets of TIICs, including B cells, CD4+ T cells, CD8+ T cells, macrophages, neutrophils and dendritic cells [28]. Here, we investigated the significance of MEX3A-D mRNA expression and the invasion of six immune cell types in lung adenocarcinoma (LUAD) and lung squamous cell carcinoma (LUSC).

## Results

**Fig. 1:**
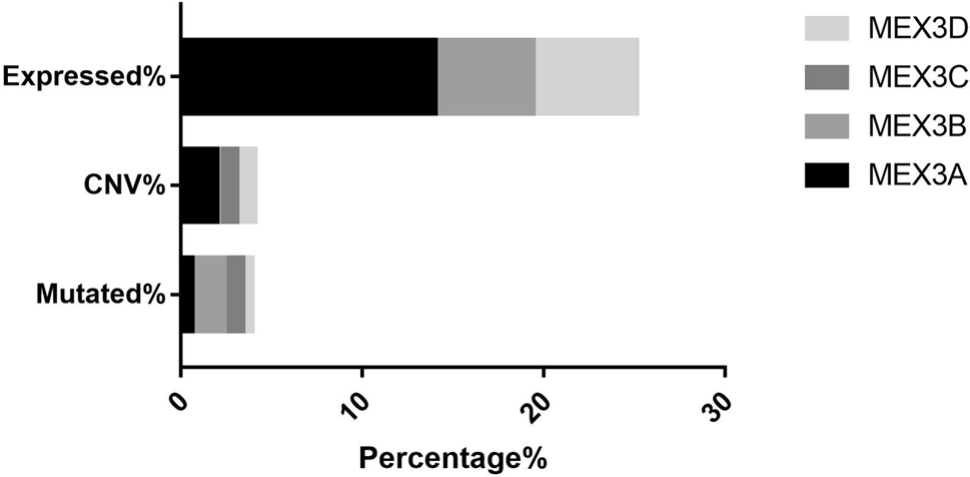
The MEX3 mutation percentage of different mutation types in lung cancer based on COSMIC database. *MEX3* muscle excess3, *CNV* copy number variation

Four MEX3 genes were assessed for mutations using the COSMIC database. Data submitted prior to February 20, 2021, were collected. Table 2 shows the highest percentage of mutation samples for each major tissue type in the COSMIC database for the MEX3 gene, including point mutations, CNV data and gene expression data and genetic alterations in *MEX3* in lung cancer samples are shown in Table 3. We found that the main regulatory mechanism in tumours, including lung cancer, was missense mutation. Furthermore, in a study on the regulatory mechanism in lung cancer, three mechanisms were found (Fig. 1): point mutations, copy number variations (CNVs), and gene expression. Of these regulatory mechanisms, point mutations mainly occurred in *MEX3B*, with the highest mutation frequency of 1.06%; CNVs in *MEX3A* were confirmed, with the highest mutation frequency of 1.99%; and *MEX3A* was determined to be overexpressed, with the highest frequency of 14.03%. No translocations, insertions, deletions, or loss of heterozygosity were identified. In summary, the results indicate that the sequence and copy number of *MEX3* genes were not altered significantly, except for *MEX3A* overexpression, which revealed that this gene family is stable and not readily mutated, leading to the malignant proliferation of tumour cells. These findings may explain the development of malignant tumours.

**Table 2:** The characteristics of *MEX3* family in COSMIC

	Chromosomal location	The distribution of mutations across the primary tissue types			Drug resistance
		Point mutations	Copy number variation	Gene expression	
*MEX3A*	1q22	Endometrium (3.09%)	Biliary tract (8.33%)	Adrenal gland (30.38%)	None
*MEX3B*	15q25.2	Large intestine (4.08%)	Skin (1.02%)	Urinary tract (9.06%)	
*MEX3C*	18q21.1	Central nervous system (6.72%)	Stomach (3.39%)	None	
*MEX3D*	19p13.3	Cervix (2.45%)	Ovary (1.32%)	Adrenal gland (Over-expressed 49.37%; Under-expressed 37.97)	

*MEX3* muscle excess 3

**Table 3:** Genetic alteration affecting *MEX3* family in lung cancer samples (COSMIC database)

	Percent of mutated samples (number)	Genetic alteration	Number	Percentage (%)
*MEX3A*	272/38263	Ponit Mutations	16/2608	0.61
		Copy number Variation	20/1006	1.99
		Gene Expression	143/1019	14.03
*MEX3B*	379/38350	Ponit Mutations	46/2609	1.76
		Copy number Variation	1/1006	0.10
		Gene Expression	55/1019	5.40
*MEX3C*	311/38693	Ponit Mutations	29/2742	1.06
		Copy number Variation	2/1006	0.99
		Gene Expression (over)	53/1019	5.20
		Gene Expression (under)	21/1019	2.06
*MEX3D*	262/38693	Ponit Mutations	13/2742	0.47
		Copy number Variation	10/1006	0.99
		Gene Expression	58/1019	5.69

*MEX3* muscle excess 3

**Fig. 2:**
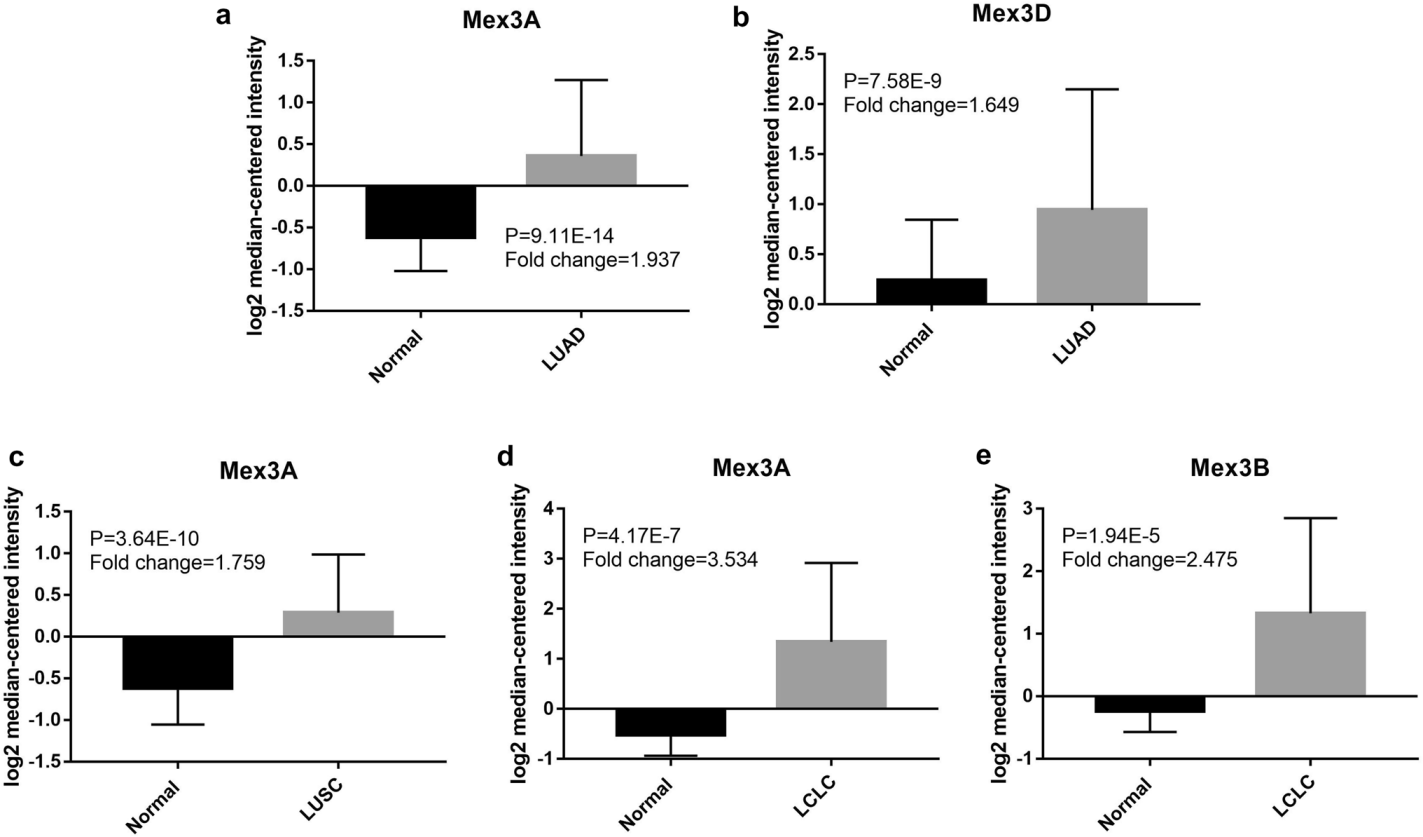
The analysis of MEX3 in lung cancer from Oncomine analysis. Column bar graph derived from gene expression data in Oncomine comparing expression levels of MEX3 in normal (left plot) and cancer (right plot) tissue and plotted using Graphpad Prism 7 software. *Y*-axis represents Mean with Standard Deviation (M ± SD). *MEX3* muscle excess 3, *LUAD* lung adenocarcinoma, *LUSC* lung squamous cell lung carcinoma, *LCLC* large cell lung carcinoma

The Oncomine™ database analysis revealed MEX3 expression in tissues of NSCLC patients compared to normal tissues. The column graph in Fig. 2 was derived from the expression of each gene in tumours of different pathological types. The analysis demonstrated that compared with normal tissues, different pathological tissues showed significant overexpression of MEX3 mRNA. It was concluded that MEX3C did not meet the criteria, but the other subtypes were overexpressed. MEX3A [29] was expressed in LUAD, LUSC and large cell lung cancer (LCLC); MEX3B [29] was detected in LCLC; and MEX3D [30] was expressed in LUAD. Furthermore, the differences in MEX3 expression based on pathological type and in different databases are summarized in Table 4.

**Table 4:** Expression of MEX3 family in different NSCLC pathological types

MEX3	Fold change	Dataset	Sample number		Total	P value
			Normal	Cancer		
A: Lung adenocarcinoma vs. normal						
EX3A	1.937	Hou Lung	65	45	110	9.11 × 10^–14^
MEX3D	1.649	Landi Lung	49	58	107	7.58 × 10^–9^
B: Squamous cell lung carcinoma vs. normal						
MEX3A	1.759	Hou Lung	65	27	92	3.64 × 10^–10^
C: Large cell lung carcinoma vs. normal						
MEX3A	3.534	Hou Lung	65	19	84	4.17 × 10^–7^
MEX3B	2.475	Hou Lung	65	19	84	1.94 × 10^–5^

*MEX3* muscle excess3, *NSCLC* not-small-cell lung cancer

**Fig. 3:**
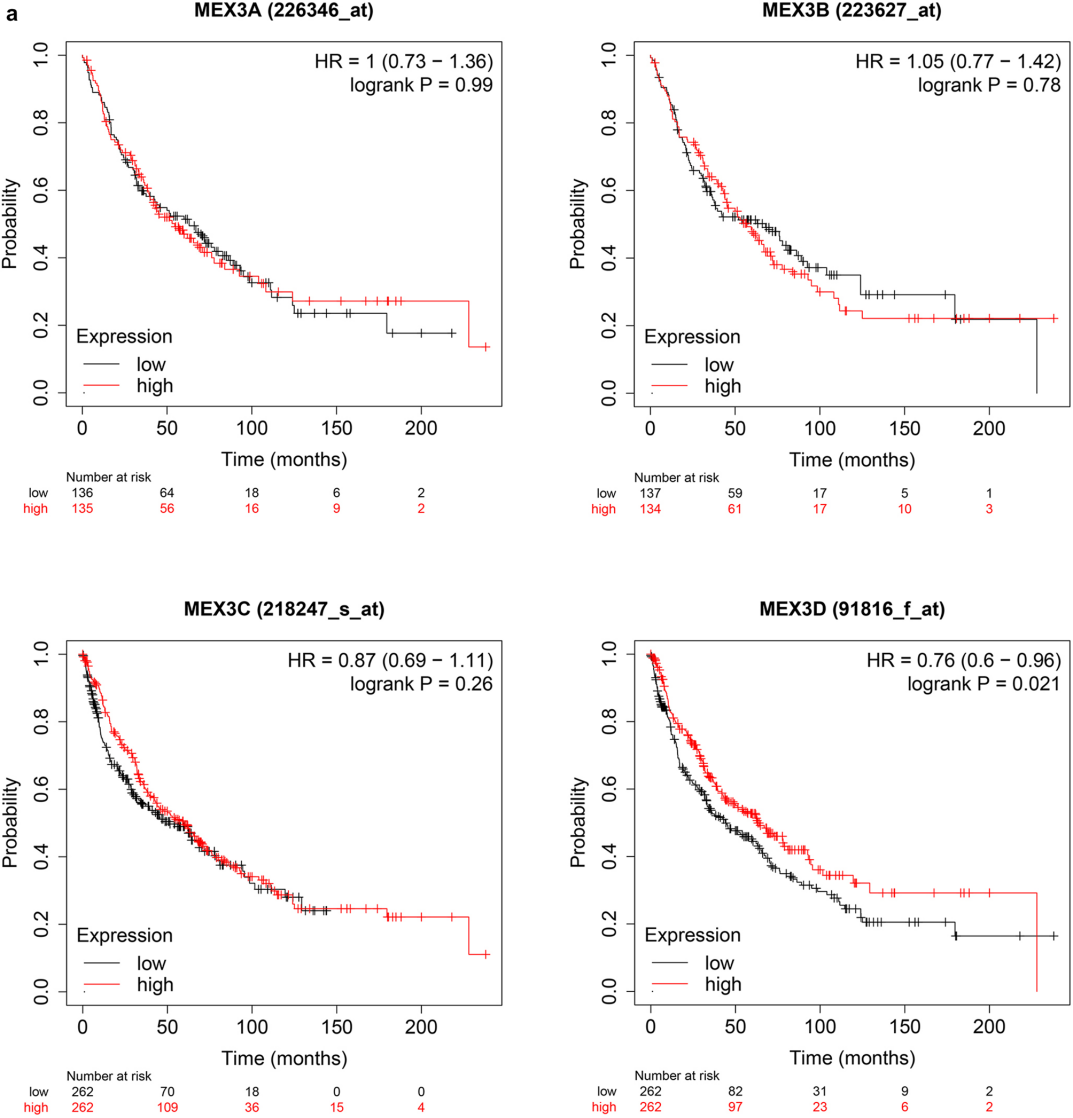

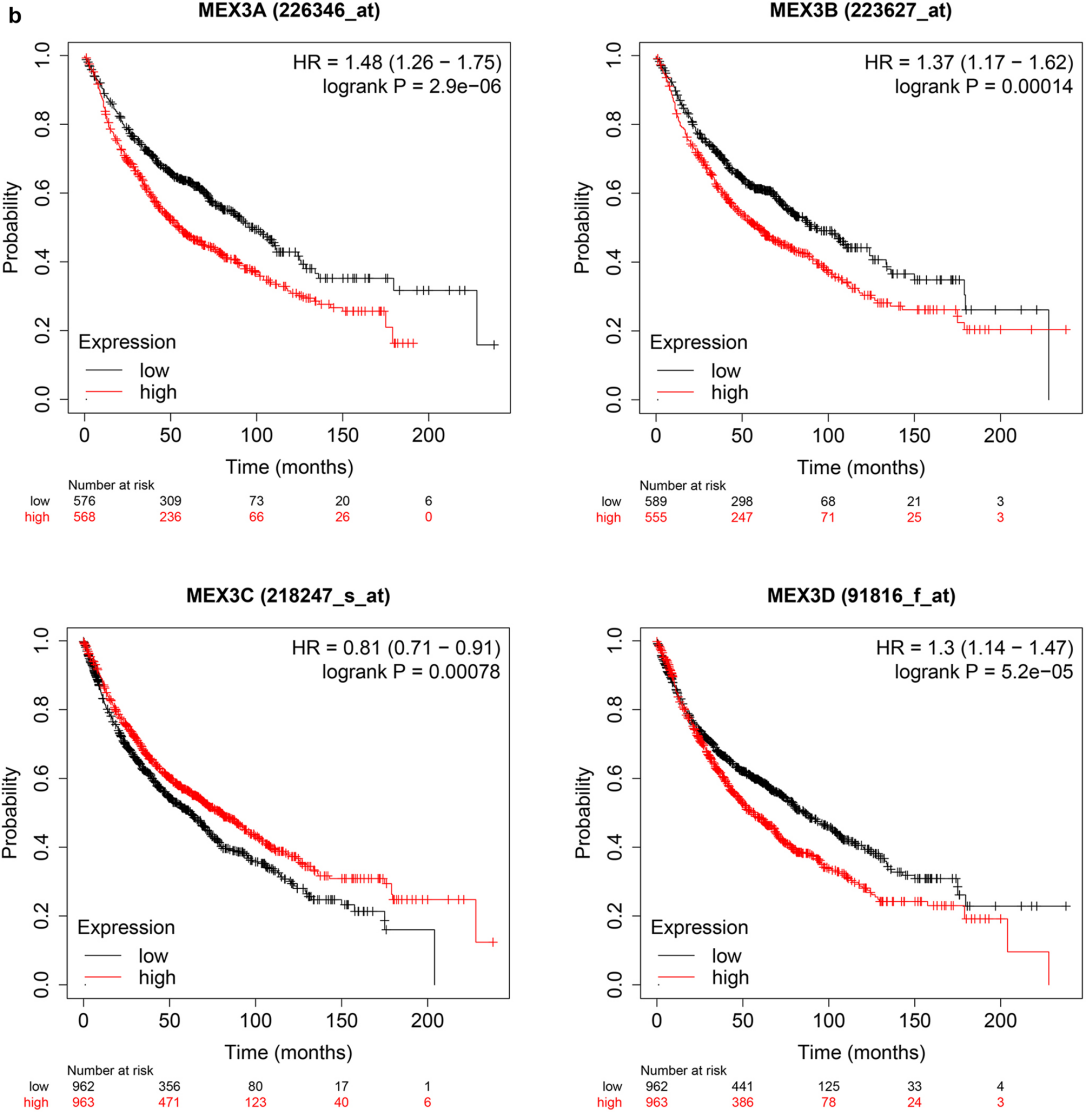

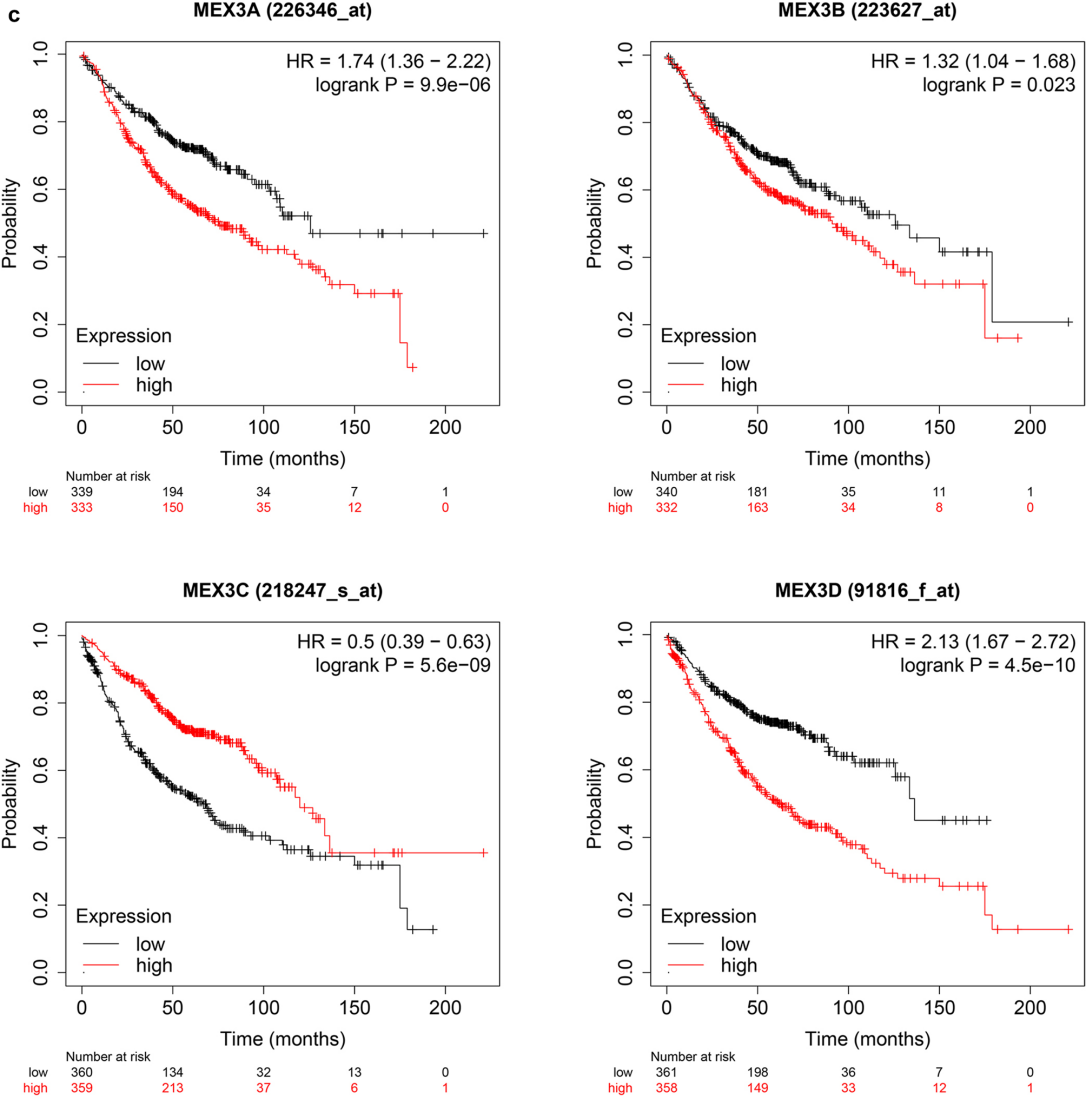
**a** The prognostic value of MEX3 expression in all NSCLC. Survival curves were plotted for all NSCLC patients (MEX3A, *n* = 1144; MEX3B, *n* = 1924; MEX3C, *n* = 1924; MEX3D, *n* = 1925). Data was analyzed using Kaplan–Meier Plotter. Patients with expression above the median are indicated in red line, and patients with expressions below the median in black line. The prognostic value of MEX3 expression in LUAD. Survival curves were plotted for LUAD patients (MEX3A, *n* = 672; MEX3B, *n* = 672; MEX3C, *n* = 719; MEX3D, *n* = 719). Data was analyzed using Kaplan–Meier Plotter. Patients with expression above the median are indicated in red line, and patients with expressions below the median in black line. **c** The prognostic value of MEX3 expression in LUSC. Survival curves were plotted for LUSC patients (MEX3A, *n* = 271; MEX3B, *n* = 271; MEX3C, *n* = 524; MEX3D, *n* = 524). Data was analyzed using Kaplan–Meier Plotter. Patients with expression above the median are indicated in red line, and patients with expressions below the median in black line. *HR* hazard ratio, *CI* confidence interval

The prognostic value of MEX3 mRNA expression was examined by Kaplan–Meier plotter [Lung Cancer] 2015 version. First, Fig. 3a demonstrates the prognostic effect of MEX3 mRNA expression in NSCLC. Overexpression of MEX3A, MEX3B and MEX3D were associated with a significantly worse prognosis for NSCLC patients: MEX3A (HR = 1.48; CI 1.26–1.75; *P* = 2.9E−06), MEX3B (HR = 1.37; CI 1.17–1.62; *P* = 1.4E−04) and MEX3D (HR = 1.30; CI 1.14–1.47; *P* = 5.2E−05). But in MEX3C, the overexpressed had better prognosis (HR = 0.81; CI 0.71–0.91; *P* = 7.8E−04). Second, the effect of MEX3 mRNA expression on prognosis was examined in LUAD are shown in Fig. 3b. Similar to NSCLC, overexpression of MEX3A, MEX3B and MEX3D were associated with decreased OS for MEX3A (HR = 1.74; CI 1.36–2.22; *P* = 9.9E−06), MEX3B (HR = 1.32; CI 1.04–1.68; *P* = 0.023) and MEX3D (HR = 2.13; CI 1.67–2.72; *P* = 4.5E−10), but not in MEX3C (HR = 0.50; CI 0.39–0.63; *P* = 5.6E−09). As shown in Fig. 3c, the prognostic value of MEX3 mRNA expression was analysed in LUSC, only in MEX3D (HR = 0.76; CI 0.60–0.96; *P* = 0.02), overexpression was related to decreased OS, but this finding was not true for patients with MEX3A (HR = 1.00; CI 0.73–1.36; *P* = 0.99), MEX3B (HR = 1.05; CI 0.77–1.42; *P* = 0.78) and MEX3C (HR = 0.87; CI 0.69–1.11; *P* = 0.26).

The associations between MEX3 and clinicopathological characteristics of NSCLC patients, including pathological histology, stage, American Joint Committee on Cancer (AJCC) T classification, lymph node status (AJCC N classification), sex, smoking status and chemotherapy, were also explored. Grade, AJCC M classification and radiotherapy could not be examined because of invalid samples. As shown in Table 5, overexpression of MEX3A, MEX3B, MEX3C and MEX3D was associated with a significantly lower OS of NSCLC and LUAD patients, while overexpression of MEX3D was associated with significantly worse OS among patients with LUSC. Furthermore, MEX3A, MEX3C and MEX3D overexpression was associated with significantly worse OS in patients with stage I disease, while MEX3C also had a consistent associated with prognosis in those with stage II disease (Table S1). MEX3A and MEX3B were both significantly associated with classification 2, whereas MEX3A was also associated with classification 1, and MEX3B was also associated with classification 3, as shown in Table S2.

**Table 5:** Correlation of MEX3 with Pathological histology in NSCLC patients

	Pathology subtype	Case-low	Case-high	HR (95% CI)	*P* value
MEX3A	All	576	568	1.48 (1.26–1.75)	2.9E−06
	LUAD	339	333	1.74 (1.36–2.22)	9.9E−06
	LUSC	136	135	1.00 (0.73–1.36)	0.99
MEX3B	All	589	555	1.37 (1.17–1.62)	1.4E−04
	LUAD	340	332	1.32 (1.04–1.68)	0.023
	LUSC	137	134	1.05 (0.77–1.42)	0.78
MEX3C	All	962	963	0.81 (0.71–0.91)	7.8E−04
	LUAD	360	359	0.50 (0.39–0.63)	5.6E−09
	LUSC	262	262	0.87 (0.69–1.11)	0.26
MEX3D	All	962	963	1.30 (1.14–1.47)	5.2E−05
	LUAD	361	358	2.13 (1.67–2.72)	4.5E−10
	LUSC	262	262	0.76 (0.60–0.96)	0.02

*NSCLC* non-small-cell lung cancer, All stands for NSCLC, *LUAD* lung adenocarcinoma, *LUSC* lung squamous cell carcinomas, *HR* hazard ratio, *CI* confidence interval, *Cases-low/high* patient number of low/high expression of the corresponding gene

MEX3A was correlated with stage N0 (Table S3), while the four MEX3 members were correlated with sex (Table S4) among NSCLC patients. MEX3A, but not MEX3C and MEX3D, was significantly associated with smoking (Table S5). In contrast, MEX3 was not associated with prognosis in NSCLC patients with or without chemotherapy (Table S6).

**Fig. 4:**
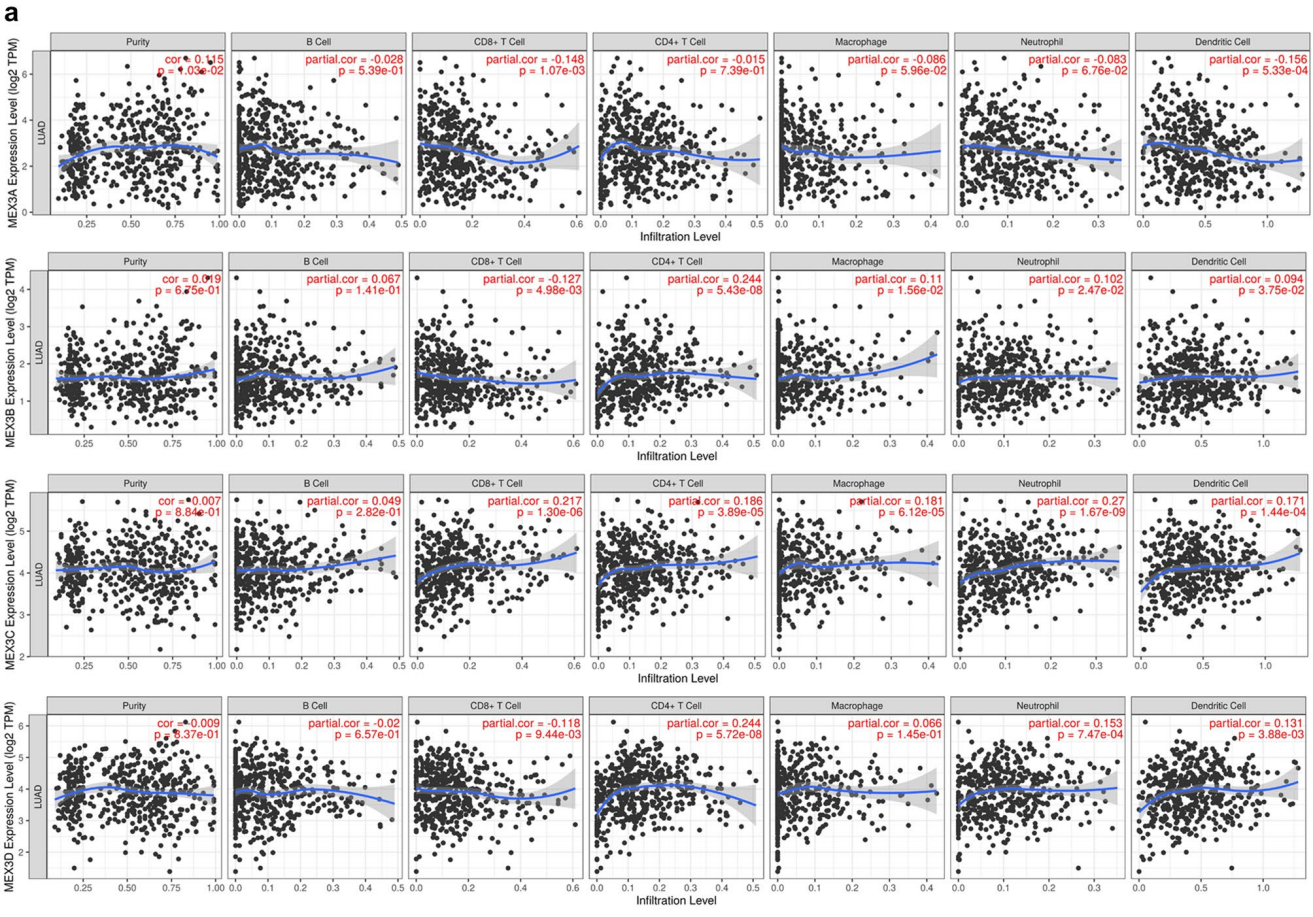

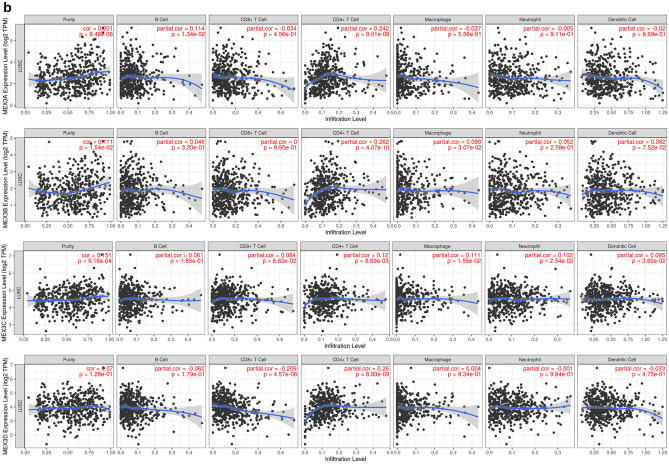
**a** Correlation between MEX3 and immune cells in LUAD. **b** Correlation between MEX3 and immune cells in LUSC

The relationship between MEX3 and the immune microenvironment of LUAD and LUSC and information on tumour purity were obtained from the TIMER 2.0 database. In LUAD, MEX3A was positively correlated with tumour purity and negatively correlated with the level of dendritic cell infiltration. MEX3B was positively correlated with tumour purity and CD4+ T cell and macrophage infiltration. MEX3C correlated not only with tumour purity but also with CD8+ T cell, CD4+ T cell, macrophage, and neutrophil infiltration. Finally, we found that MEX3D was statistically related to tumour purity and positively related to CD4+ T cells, macrophages and neutrophils (Fig. 4a). In LUSC, MEX3A was similarly shown to be positively correlated with tumour purity and B cells and negatively correlated with CD4+ T cells. MEX3B and MEX3C were positively correlated with tumour purity, CD8+ T cells and CD4+ T cells. MEX3D had no significant effect on tumour purity but was positively correlated with CD4+ T cells and macrophages (Fig. 4b). We also found that MEX3 overexpression was not significantly related to the survival rate of NSCLC patients, regardless of time point (1, 3 or 5 years) (*P* > 0.05).

## Discussion

The human MEX3 family is differentially expressed in healthy tissues of different origins [12], so we were interested in understanding how these family members are expressed in abnormal tissues, particularly in cancer. The Human Protein Atlas shows that members of the MEX3 family are expressed in heterogeneous types of tumours [31, 32]. Many studies have indicated that MEX3A promotes cell proliferation and inhibits cell apoptosis in bladder cancer [33, 34], gastric cancer [35], and colorectal cancer [36]. Furthermore, increased MEX3A levels were also reported in liver cancer and were shown to be significantly associated with worse patient survival [37]. This study revealed that the overexpression of MEX3A conferred a significantly worse prognosis in NSCLC and LUAD but not LUSC. In addition, increased expression of MEX3A in NSCLC patients was associated with stage I tumour and lymph node status and male sex, and chemotherapy had little effect on this prognosis. There are few studies on the role of MEX3B in cancer. This protein may ubiquitinate Runx3 (runt-related transcription factor 3) and increase the invasion of gastric cancer cells [38]. Our study revealed that high expression of MEX3B mRNA was associated with decreased OS in all NSCLC and LUAD cases. MEX3B overexpression was also associated with stage T2 and T3 and the sex of NSCLC patients.

Recently, the MEX3A and MEX3C proteins were reported to be negative posttranslational regulators of several target genes [39]. In colorectal cancer, MEX3C has been identified as an unstable gene that is frequently lost in CIN+ (cervical intraepithelial neoplasia+) [40, 41], and this protein was shown to regulate lipid metabolism through the JNK (c-Jun N-terminal kinase) pathway in bladder cancer [42] and breast cancer [43]. Based on these observations, we hypothesized that MEX3C plays an important role in influencing metastasis and prognosis in NSCLC. Consistent with our findings, other studies have shown that high MEX3C mRNA expression is associated with poor prognosis (OS) in NSCLC and LUAD patients. Several studies [44, 45] demonstrated that MEX3D reverses apoptosis by interacting with au-rich elements (AREs) and enhances the degradation of BCL2 (B cell lymphoma 2) mRNA. Moreover, MEX3D is frequently deleted in various human cancers [46], participates in modulating the effectiveness of chemotherapy in AML (acute myeloid leukaemia) [47], and is overexpressed in androgen-independent prostate cancer [48]. In the Kaplan–Meier analysis in this study, high expression of MEX3D was observed and found to be a good prognostic indicator in LUAD, LUSC and all NSCLC.

In the immune response, MEX3B can function as a coreceptor in the innate antiviral response of Toll-like receptors [49]. In melanoma [50], MEX3B downregulation is associated with antibodies against programmed cell death 1 (PD-1), while overexpression of this protein can inhibit T cell-mediated tumour elimination. MEX3C is involved in the regulation of proteins via degradation and ubiquitination, and it has been identified as a new type of RNA-binding E3 ubiquitin ligase that is responsible for posttranscriptional regulation [51]. The results of the present study showed that MEX3B and MEX3C expression was positively related to tumour purity and CD8+ T cell and CD4+ T cell infiltration. However, the function of MEX3A and MEX3D in immune responses has not been sufficiently researched. Abundant evidence [11, 12] indicates that MEX3 proteins have the ability to regulate gene expression with a negative correlation with tumour suppressors. In follow-up studies, we plan to explore targeted inhibitors of MEX3 and to determine if MEX3 is a marker for immunotherapy.

## Conclusion

In summary, research using the COSMIC database revealed no major alterations in the sequence or copy number of MEX, which can explain its higher copy number in malignant tumours and its correlation with malignant proliferation. We also used the Oncomine™ database to determine the expression of MEX3 in NSCLC, and Kaplan–Meier analysis was used to ascertain the prognostic value of these genes. The results indicated that in NSCLC and LUAD, MEX3A, MEX3B, MEX3C, and MEX3D overexpression was significantly associated with a worse prognosis, and MEX3D overexpression was also associated with poor OS in LUSC. In addition, TIMER analysis revealed that MEX3B and MEX3C were positively related to tumour purity and CD8+ T cell and CD4+ T cell infiltration.

These data reflect the potential association of MEX3 with non-small-cell lung cancer. We found that most MEX3 family members are highly expressed in NSCLC. High expression indicates a poor prognosis and has certain correlations with immune cell infiltration. Therefore, these conclusions lay the framework for determining the prognosis of NSCLC patients and developing novel treatment strategies in the future. However, of course, our research needs to be improved and extended. In the future, we need to further analyse the posttranscriptional regulatory mechanisms and immunomodulatory effects of MEX3, given the current focus on tumour immunotherapy.

## Limitations

This study only collected and analysed correlated expression in databases at the genetic level and was not validated in tissue or clinical studies in non-small cell lung cancer, which is our biggest shortcoming. Secondly, a more refined analysis of the expression and immune correlation of one of the genes in lung adenocarcinoma or squamous carcinoma could have been performed to highlight the core ideas of the article without being repetitive and lengthy. But it is these limitations that make us even more motivated to explore further, and our team will work together to continue to explore these topics at the tissue and clinical level.

## Supplementary Information

Below is the link to the electronic supplementary material.

Supplementary file1 (DOCX 27 KB)

## Data Availability

The datasets analysed in the present study are available in the COSMIC database (https://cancer.sanger.ac.uk/cosmic) and Oncomine™ database (https://www.oncomine.org/resource) and the Kaplan Meier plotter [Lung Cancer] (http://kmplot.com/analysis/index.php?p=service&cancer=lung) and the TMIER (http://timer.comp-genomics.org/).
