# Supplementary material for: Investigation of the Potential Correlation Between RNA-Binding Proteins in the Evolutionarily Conserved MEX3 Family and Non-small-Cell Lung Cancer
Source: Mol Biotechnol. 2022 Dec 12;65(8):1263–74. doi: 10.1007/s12033-022-00638-2 (PMC10352443; doi:10.1007/s12033-022-00638-2)
Supplement: Supplementary file 1 — Supplementary file1 (DOCX 27 KB) [file 12033_2022_638_MOESM1_ESM.docx]

Table S1. Correlation of Mex3 with Stage of NSCLC patients.

| Gene | Stage | Case-low | Case-high | HR(95%CI) | logrank P |
| --- | --- | --- | --- | --- | --- |
| Mex3A | I | 225 | 224 | 1.79(1.29-2.47) | 3.6E-04 |
|  | II | 80 | 81 | 0.79(0.50-1.24) | 0.31 |
|  | III | 22 | 22 | 0.69(0.35-1.38) | 0.29 |
| Mex3B | I | 229 | 220 | 1.20(0.88-1.64) | 0.25 |
|  | II | 80 | 81 | 0.99(0.63-1.55) | 0.95 |
|  | III | 22 | 22 | 1.32(0.66-2.64) | 0.42 |
| Mex3C | I | 288 | 289 | 0.52(0.40-0.69) | 2.1E-06 |
|  | II | 122 | 122 | 0.54(0.37-0.78) | 8.7E-04 |
|  | III | 35 | 35 | 1.29(0.75-2.23) | 0.36 |
| Mex3D | I | 288 | 289 | 1.82(1.38-2.41) | 2.0E-05 |
|  | II | 123 | 121 | 1.15(0.80-1.66) | 0.45 |
|  | III | 35 | 35 | 0.79(0.45-1.36) | 0.39 |

Abbreviation: NSCLC, non-small-cell lung cancer; HR: hazard ratio; CI: confidence interval; Cases-low/high: patient number of low/high expression of the corresponding gene.

Table S2. Correlation of Mex3 with AJCC stage T of NSCLC patients.

| Gene | T | Case-low | Case-high | HR(95%CI) | logrank P |
| --- | --- | --- | --- | --- | --- |
| Mex3A | 1 | 115 | 109 | 1.53(1.02-2.28) | 0.036 |
|  | 2 | 95 | 95 | 1.47(1.01-2.15) | 0.045 |
|  | 3 | 14 | 15 | 2.09(0.93-4.72) | 0.071 |
|  | 4 | 12 | 11 | 1.91(0.82-4.46) | 0.130 |
| Mex3B | 1 | 117 | 107 | 0.86(0.58-1.27) | 0.45 |
|  | 2 | 99 | 91 | 1.69(1.15-2.48) | 6.5E-03 |
|  | 3 | 14 | 15 | 2.94(1.24-7.00) | 0.011 |
|  | 4 | 12 | 11 | 0.77(0.32-1.83) | 0.550 |
| Mex3C | 1 | 218 | 219 | 0.80(0.60-1.06) | 0.12 |
|  | 2 | 294 | 295 | 1.23(0.99-1.54) | 0.062 |
|  | 3 | 40 | 41 | 1.08(0.65-1.79) | 0.77 |
|  | 4 | 23 | 23 | 1.28(0.68-2.40) | 0.45 |
| Mex3D | 1 | 218 | 219 | 0.99(0.75-1.32) | 0.96 |
|  | 2 | 294 | 295 | 0.93(0.74-1.16) | 0.52 |
|  | 3 | 40 | 41 | 1.00(0.61-1.66) | 0.99 |
|  | 4 | 23 | 23 | 0.69(0.37-1.28) | 0.23 |

Abbreviation: AJCC, American Joint Committee on Cancer; NSCLC, non-small-cell lung cancer; HR: hazard ratio; CI: confidence interval; Cases-low/high: patient number of low/high expression of the corresponding gene.

Table S3. Correlation of Mex3 with lymph node status (AJCC N) of NSCLC patients.

| Gene | Lymph node status | Case-low | Case-high | HR(95%CI) | logrank P |
| --- | --- | --- | --- | --- | --- |
| Mex3A | 0 | 162 | 162 | 1.60(1.16-2.20) | 3.6E-03 |
|  | 1 | 51 | 51 | 1.35(0.83-2.22) | 0.23 |
|  | 2 | 16 | 16 | 1.10(0.53-2.29) | 0.80 |
| Mex3B | 0 | 164 | 160 | 1.01(0.74-1.38) | 0.95 |
|  | 1 | 52 | 50 | 1.25(0.76-2.05) | 0.39 |
|  | 2 | 16 | 16 | 1.37(0.66-2.87) | 0.40 |
| Mex3C | 0 | 391 | 390 | 1.13(0.92-1.40) | 0.24 |
|  | 1 | 126 | 126 | 0.87(0.64-1.20) | 0.40 |
|  | 2 | 56 | 55 | 1.12(0.75-1.69) | 0.57 |
| Mex3D | 0 | 391 | 390 | 1.13(0.92-1.40) | 0.25 |
|  | 1 | 126 | 126 | 0.78(0.57-1.07) | 0.12 |
|  | 2 | 56 | 55 | 1.03(0.69-1.54) | 0.89 |

Abbreviation: NSCLC, non-small-cell lung cancer; HR: hazard ratio; CI: confidence interval; Cases-low/high: patient number of low/high expression of the corresponding gene.

Table S4. Correlation of Mex3 with gender of NSCLC patients.

| Gene | Gender | Case-low | Case-high | HR(95%CI) | logrank P |
| --- | --- | --- | --- | --- | --- |
| Mex3A | female | 187 | 187 | 1.38(0.98-1.94) | 0.06 |
|  | male | 330 | 329 | 1.31(1.07-1.61) | 8.5E-03 |
| Mex3B | female | 189 | 185 | 1.19(0.85-1.68) | 0.30 |
|  | male | 332 | 327 | 1.26(1.02-1.54) | 0.028 |
| Mex3C | female | 357 | 357 | 0.69(0.55-0.88) | 1.9E-03 |
|  | male | 550 | 550 | 0.88(0.75-1.03) | 0.11 |
| Mex3D | female | 358 | 356 | 1.49(1.18-1.89) | 7.5E-04 |
|  | male | 550 | 550 | 1.17(1.00-1.38) | 0.045 |

Abbreviation: NSCLC, non-small-cell lung cancer; HR: hazard ratio; CI: confidence interval; Cases-low/high: patient number of low/high expression of the corresponding gene.

Table S5. Correlation of Mex3 with smoking status of NSCLC patients.

| Gene | Smoking status | Case-low | Case-high | HR(95%CI) | logrank P |
| --- | --- | --- | --- | --- | --- |
| Mex3A | Smoked | 150 | 150 | 1.80(1.19-2.73) | 4.9E-03 |
|  | Never smoked | 70 | 71 | 1.31(0.58-2.95) | 0.52 |
| Mex3B | Smoked | 151 | 149 | 1.05(0.70-1.57) | 0.83 |
|  | Never smoked | 72 | 69 | 1.54(0.68-3.47) | 0.29 |
| Mex3C | Smoked | 410 | 410 | 0.83(0.67-1.02) | 0.07 |
|  | Never smoked | 102 | 103 | 0.27(0.14-0.51) | 1.7E-05 |
| Mex3D | Smoked | 411 | 409 | 1.22(0.99-1.50) | 0.063 |
|  | Never smoked | 102 | 103 | 4.48(2.29-8.77) | 1.6E-06 |

Abbreviation: NSCLC, non-small-cell lung cancer; HR: hazard ratio; CI: confidence interval; Cases-low/high: patient number of low/high expression of the corresponding gene.

Table S6. Correlation of Mex3 with Chemotherapy of NSCLC patients.

| Gene | Chemotherapy | Case-low | Case-high | HR(95%CI) | logrank P |
| --- | --- | --- | --- | --- | --- |
| Mex3A | No | 10 | 11 | 1.6*10^9(0.0-Inf) | 0.014# |
|  | Yes | 17 | 17 | 0.71(0.23-2.24) | 0.56 |
| Mex3B | No | 10 | 11 | 0.87(0.17-4.32) | 0.86 |
|  | Yes | 17 | 17 | 0.83(0.26-2.59) | 0.75 |
| Mex3C | No | 156 | 154 | 0.81(0.58-1.13) | 0.20 |
|  | Yes | 88 | 88 | 1.07(0.71-1.61) | 0.76 |
| Mex3D | No | 155 | 155 | 1.16(0.83-1.63) | 0.38 |
|  | Yes | 88 | 88 | 0.97(0.64-1.46) | 0.87 |

Abbreviation: NSCLC, non-small-cell lung cancer; HR: hazard ratio; CI: confidence interval; Cases-low/high: patient number of low/high expression of the corresponding gene. #Influence of sample size, HR value is abnormal, P value is not adopted.
